# Increased variability of mean arterial pressure is associated with increased risk of short-term mortality in intensive care unit: A retrospective study

**DOI:** 10.3389/fneur.2022.999540

**Published:** 2022-09-29

**Authors:** Jia Yao, Dandan Liu, Weifeng Huang, Yuexin Fang, Yifan Yang, Yingchuan Li, Pengyuan Liu, Xiaoqing Pan

**Affiliations:** ^1^Department of Mathematics, Shanghai Normal University, Shanghai, China; ^2^Department of Critical Care Medicine, Shanghai Sixth People's Hospital, Shanghai Jiaotong University, Shanghai, China; ^3^Department of Respiratory Medicine, Sir Run Run Shaw Hospital and Institute of Translational Medicine, Zhejiang University School of Medicine, Hangzhou, China

**Keywords:** mean arterial pressure (MAP), short-term mortality, intensive care unit (ICU), odds ratio (OR), coefficient of variation

## Abstract

**Background:**

In intensive care unit (ICU), what thresholds of MAP variability are effective in distinguishing low- and high-risk patients for short-term mortality (in-hospital and 28-day) remains unclear.

**Methods:**

Fifteen thousand five hundred sixty adult subjects admitted to ICU at Beth Israel Deaconess Medical Center (Boston, USA) between 2001 and 2012 were included in this retrospective study from MIMIC-III database. MAP within the first 24 h after admission were collected. Quantiles of MAP variability from 10% to 90% with 10% increasement each were considered to divide study participants into two groups, either having coefficients of variation of MAP greater or less than the given threshold. The threshold of MAP variability was identified by maximizing the odds ratio associated with increased risk of short-term mortality (in-hospital and 28-day). Logistic regression and Cox regression models were further applied to evaluate the association between increased variability of MAP and short-term mortality (in-hospital and 28-day).

**Results:**

90% quantile of MAP variability was determined as the threshold generating the largest odds ratio associated with the increased risk of short-term mortality. Increased MAP variability, especially over 90% of MAP variability, was associated with increased risk of in-hospital mortality (odds ratio: 2.351, 95% CI: 2.064–2.673), and 28-day mortality (hazard ratio: 2.064, 95% CI: 1.820–2.337).

**Conclusion:**

Increased MAP variability, especially over 90% of MAP variability, is associated with short-term mortality. Our proposed threshold of MAP variability may aid in the early identification of critically ill patients with a high risk of mortality.

## Introduction

Blood pressure (BP) is a fundamental physiological variable monitored in intensive care medicine. The variation of BP arises naturally because BP is influenced by biological, behavioral, emotional, and environmental factors and their complex interactions (1–5). The increased variation of BP has been reported to be associated with various organ injuries, high risks of cardiovascular and cerebrovascular events, and mortality, as it reflects sympathetic activation and impairment of baroceptive reflexes (6–9).

BP variation is a continuous phenotype, mainly divided into short-term (minutes to hours) and long-term (days and months) variation. Both short- and long-term blood pressure variability independently increased the risk of death in hypertensive patients as well as in patients with diabetes and chronic kidney disease (10–12). Critically ill patients are often accompanied by high incidence of anxiety, delirium, sleep deprivation, central, and autonomic dysregulation during intensive care unit (ICU) (13), which may contribute to increased BP variation, especially short-term BP variation. Increased short-term BP variability is known to adversely affect patients with chronic diseases, however the extent to which increased short-term BP variability increases the risk of in-hospital mortality in critically ill patients in the ICU remains to be further investigated.

Circadian rhythm of mean arterial pressure (MAP) is recommended for assessing the prognosis of patients admitted to ICU in the clinical setting (14, 15). Although accumulating evidence also indicated that increased BP variability was independently associated with higher risk of target-organ damage, cardiovascular event, and mortality (11, 13, 16–18), little was known about the threshold at which MAP variability is high enough to have clinical significance in critically ill patients. In this study, we hypothesized that increased MAP variability is associated with short-term mortality and further proposed a threshold of MAP variability to aid early identification of critically ill patients at a high risk of mortality.

## Methods

### Study participants

Patients admitted to ICU at Beth Israel Deaconess Medical Center (Boston, USA) between 2001 and 2012 were included in this retrospective study from Multiparameter Intelligent Monitoring for Intensive Care (MIMIC)-III database (v 1.4) (19). The database was approved by the Institutional Review Boards of Beth Israel Deaconess Medical Center (Boston, MA) and the Massachusetts Institute of Technology (Cambridge, MA).

In this study, we mainly included adult patients, with blood pressure records in the first 24 hours after admission. The exclusion criteria were (1) patients younger than 18 years or older than 89 years, (2) multiple ICU admissions or hospitalizations, (3) ICU admission less than one day, (4) vasoactive and sedative medication usage on the first day of admission, (5) < 2 observations of concurrently measured invasive or non-invasive blood pressure.

### Study data

Demographic and phenotypic data extracted from MIMIC-III database included: age, sex, ethnicity, length of ICU stay, the Sequential Organ Failure Assessment (SOFA) score, systolic blood pressure (SBP) and diastolic blood pressure (DBP), diseases classification using ICD-9, outcomes (in-hospital and 28-days mortality), ventilation (a categorical variable, describing a presence of artificial ventilation or not), the vasopressor medications and sedatives as described in the previous study (20). Here, the SOFA score measures the aggregate severity of organ dysfunction in six organ systems (respiratory, coagulatory, liver, cardiovascular, renal, and neurologic) (21–23). MAP was calculated as MAP = (SBP+2 × DBP)/3.

Each patient had at least two BP measurements with an average 20 BP measurements. For each patient, mean and standard deviation were calculated from multiple BP measurements. The coefficient of variation (CV), calculated as the standard deviation divided by the mean, was applied to measure the variability of MAP. Quantiles of MAP variability from 10% to 90% with 10% increasement each were considered to divide study participants into two groups: high variability (≥ each quantile of CV) and low variability (< each quantile of CV).

### Statistical analysis

For the demographic and clinical data collected in this study, age was reported using median and interquartile range (IQR). BP-related variables were described using mean, standard deviation and the range between minimum and maximum of BP. Categorical variables were described using frequencies and percentages.

Two-sample *t*-test or Mann-Whitney *U* test was applied to compare differences between two independent groups when the corresponding variable was normally or non-normally distributed. Categorical variable was tested using the Chi-squared tests or Fisher's exact tests. Logistic regression and Cox regression models were performed to evaluate the relationship between CV of MAP and in-hospital mortality, and CV of MAP and 28-day mortality, respectively. In addition to the univariate logistic regression and Cox regression models, three models with adjustments of covariates were further considered. Model 1 was adjusted for age, sex, and ethnicity. Model 2 was adjusted for the covariates included in model 1 plus SOFA score. Model 3 was adjusted for the covariates included in models 2 plus ventilation (categorical variable, yes/no). In above models, low variability group was considered as the reference group to estimate odds ratio and hazard ratio. Kaplan-Meier survival curves of low and high variability groups were illustrated. The survival differences in 28-day mortality between the two groups were compared by the log-rank test. *P* < 0.05 was considered statistically significant. All statistical analyses were performed using R Statistical Software (version 4.1.0, https://www.r-project.org/).

## Results

### Characteristics of overall study participants

**Figure 1:**
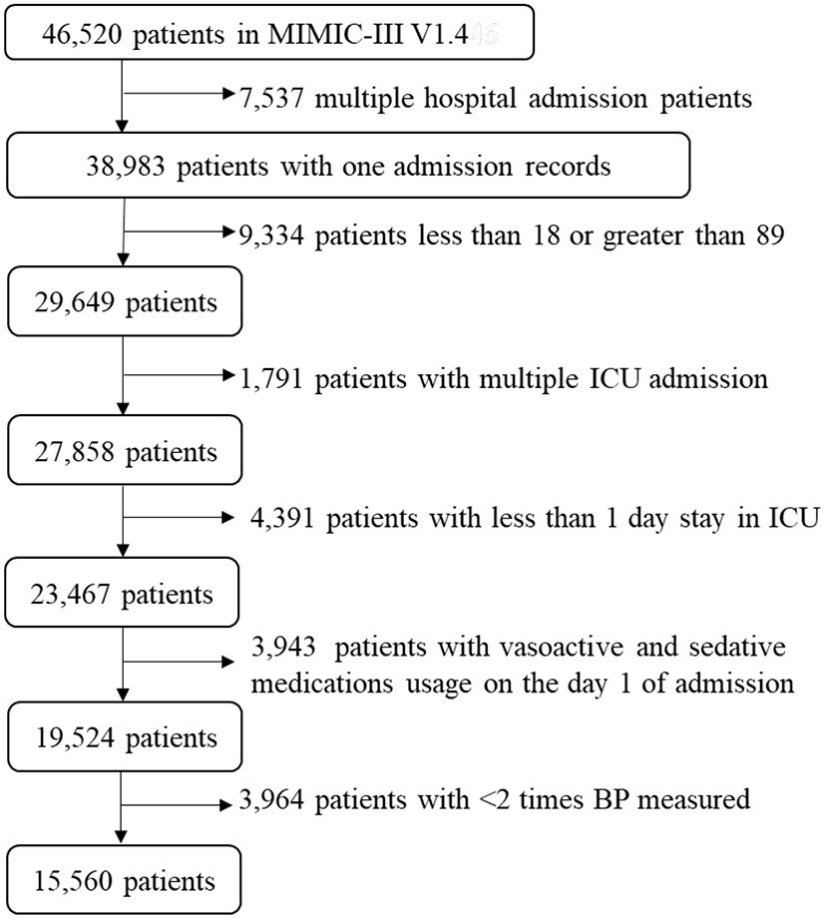
A flow chart of recruiting study participants. A total of 15,560 patients were included in this study. ICU, intensive care unit; BP, blood pressure; MIMIC-III, Multiparameter Intelligent Monitoring in Intensive Care III.

Of 46,520 subjects in MIMIC-III database, we excluded 7,537 patients with multiple hospital admissions, 9,334 patients under 18 years of age or over 89 years of age, 1,791 patients with multiple ICU admissions, 4,391 patients with < 1 day stay in ICU, 3,943 patients with vasoactive and sedative medications usage on the first day of admission, and 3,964 patients with < 2 BP measurements. Finally, 15,560 patients were retained in this study (Figure 1).

Of 15,560 patients, 57.62% were over 60 years old, the median age was 63, 56.34% were male, 68.73% were white, and 45.49% were diagnosed with hypertension at admission. The in-hospital and 28-day mortality rates were 12.37% and 14.71%. The causes of admission to ICU included hypertension, hypotension, acute myocardial Infarction and so on. Detailed characteristics of study participants are shown in Table 1. On average, patients diagnosed with coma and self-poisoning had slightly lower MAP variability than patients diagnosed with hypertension, hypotension, acute myocardial infarction and so on (Supplemental Figure 1).

**Table 1:** Characteristics of study participants.

**Characteristic, No (%)**	**Total (*n* = 15,560)**	**CV < 90% Q**	**CV≥90% Q**	***P***
		**(*n* = 14,004)**	**(*n* = 1,556)**	
**Age, median (interquartile range)**	63 (50–76)	62 (49–75)	71 (57–80)	*P* < 0.001
18–44	2,758 (17.72)	2,589 (18.49)	169 (10.86)	*P* < 0.001
45–59	3,837 (24.66)	3,556 (25.39)	281 (18.06)	
≥60	8,965 (57.62)	7,859 (56.12)	1106 (71.08)	
**Sex**
Male	8767 (56.34)	8,004 (57.16)	763 (49.04)	*P* < 0.001
Female	6793 (43.66)	6,000 (42.84)	793 (50.96)	
**Ethnicity**
White	10694 (68.73)	9628 (68.75)	1066 (68.51)	*P* = 0.745
Black	1134 (7.29)	1028 (7.34)	106 (6.81)	
Asian	402 (2.58)	362 (2.58)	40 (2.57)	
Hispanic or Latino	485 (3.12)	441 (3.15)	44 (2.83)	
Unknown/Other	2,845 (18.28)	2,545 (18.17)	300 (19.28)	
**Ventilation**	2561 (16.46)	2221 (15.86)	340 (21.85)	*P* < 0.001
**Day 1 SOFA, median (IQR)**	3 (2–5)	3 (1–5)	4 (2–7)	*P* < 0.001
**The causes of admission to the ICU (diseases by ICD-9), No (%)**
Diabetes mellitus	2,834 (18.21)	2,533 (18.09)	301 (19.34)	*P* = 0.236
Hypertension	7079 (45.49)	6363 (45.44)	716 (46.02)	*P* = 0.683
Hypotension	1305 (8.39)	1125 (8.03)	180 (11.57)	*P* < 0.001
Cerebral hemorrhage	643 (4.13)	572 (4.08)	71 (4.56)	*P* = 0.405
Shock	1520 (9.77)	1281 (9.15)	239 (15.36)	*P* < 0.001
Respiratory failure	2694 (17.31)	2299 (16.42)	395 (25.39)	*P* < 0.001
Self-poisoning	337 (2.17)	321 (2.29)	16 (1.03)	*P* = 0.002
Infection	3904 (25.09)	3389 (24.20)	515 (33.10)	*P* < 0.001
Coma	958 (6.16)	879 (6.28)	79 (5.08)	*P* = 0.070
Delirium	640 (4.11)	561 (4.01)	79 (5.08)	*P* = 0.051
Acute myocardial infarction	1,037 (6.66)	926 (6.61)	111 (7.13)	*P* = 0.466
Pulmonary embolism	409 (2.63)	377 (2.69)	32 (2.06)	*P* = 0.161
Congestive heart failure	2,968 (19.07)	2,592 (18.51)	376 (24.16)	*P* < 0.001
**Short-term mortality, No (%)**
Hospital	1925 (12.37)	1569 (11.20)	356 (22.88)	*P* < 0.001
28-day	2289 (14.71)	1907 (13.62)	382 (24.55)	*P* < 0.001
**Blood pressure, mmHg, mean (SD), min-max**
SBP	120.08 (18.22)	120.37 (18.12)	117.35 (18.95)	*P* < 0.001
	46–231	46–231	52-220	
DBP	62.17 (11.55)	62.43 (11.46)	59.80 (12.08)	*P* < 0.001
	22–157	22–126	25–157	
MAP	81.47 (12.20)	81.75 (12.11)	78.99 (12.71)	*P* < 0.001
	34–170	35–156	34–170	

SOFA, Sequential Organ Failure Assessment; IQR, interquartile range; ICD-9, International Classification of Diseases, Ninth Revision; SD, standard deviation; SBP, systolic blood pressure; DBP, diastolic blood pressure; MAP, mean arterial pressure; CV, coefficient of variation; 90% Q, 90% quantile of CV of MAP.

In addition, 3586 (23.0%) had invasive BP only, 8960 (57.6%) had non-invasive BP only, and 3014 (19.4%) had both invasive and non-invasive BP. On average, MAP variability was 0.1121, 0.1145, and 0.1144 in subjects with invasive BP only, non-invasive BP only, and both invasive and non-invasive BP, respectively. Since the MAP variability is very close between groups with invasive and non-invasive BP measurements, we did not distinguish these two BP measurements in the downstream data analysis.

### Comparison of MAP variability thresholds

**Figure 2:**
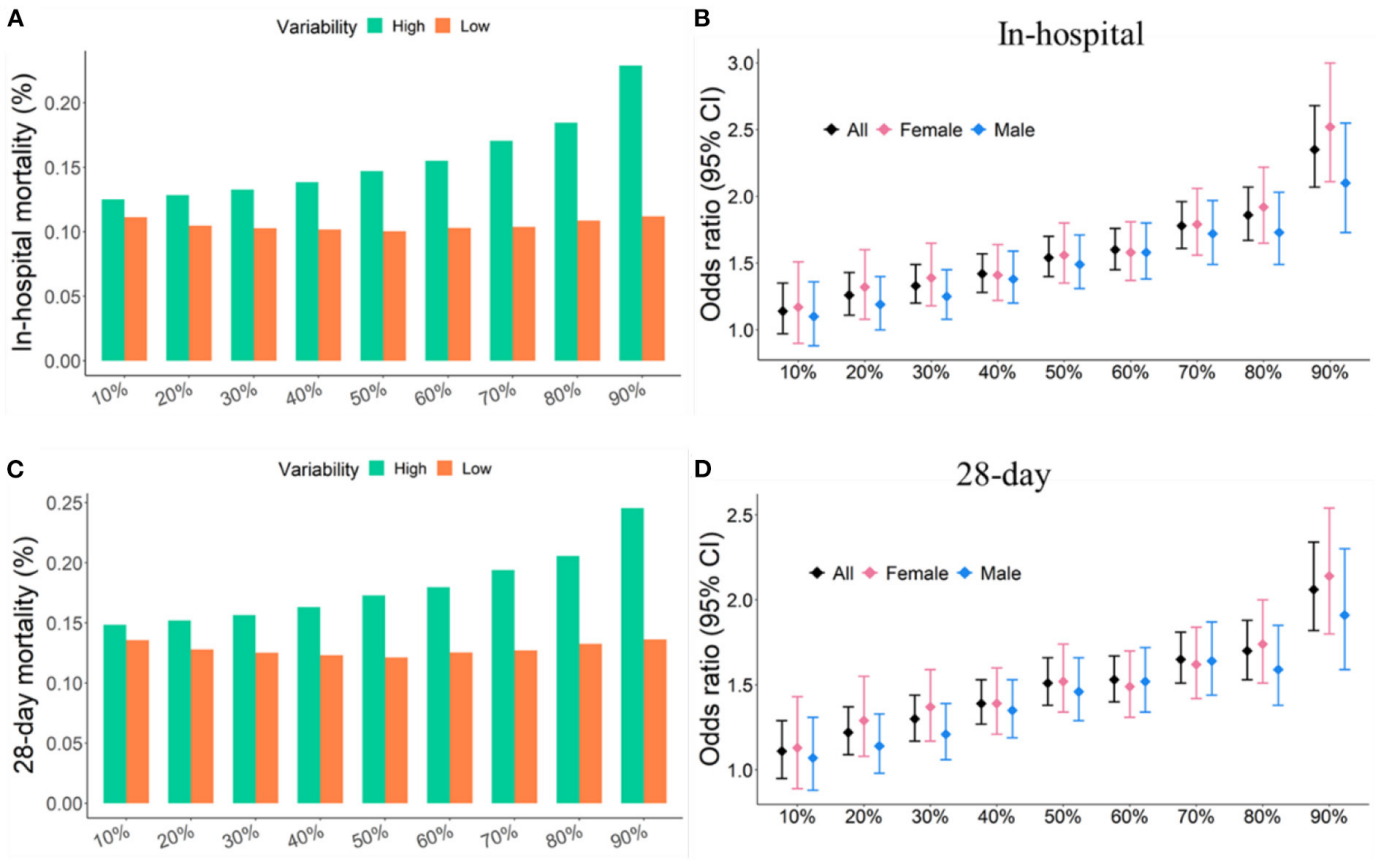
Short-term mortality and odds ratios were increased with increasing quantile of MAP variability. **(A,C)** Short-term mortality as a function of different quantiles of CV. **(B,D)** Odd ratio as a function of different quantiles of CV. Study participants were divided into two groups: high variability (≥ a given quantile of CV) and low variability (< a given quantile of CV).

Quantiles of MAP variability from 10% to 90% with 10% increasement each were considered to divide study participants into two groups: high variability (≥ each quantile of CV) and low variability (< each quantile of CV). In-hospital and 28-day mortality rates were relatively stable, about 12%, in patients with low MAP variability. However, in-hospital and 28-day mortality rates increased with increasing MAP variability in patients with high MAP variability. In addition, the odds ratios associated with the increased risk of short-term mortality also increased as the threshold of MAP variability increased. The same trend was also observed in subgroups of male and female subjects. In general, women had larger odds ratio associated with 28-day mortality than men. 90% quantile of MAP variability was determined as the threshold that generated the largest odds ratio associated with the increased risk of short-term mortality (Figure 2). The in-hospital and 28-day mortality in patients with over 90% of MAP variability were almost twice as high as those with < 90% of MAP variability (Table 1 and Figure 2).

**Figure 3:**
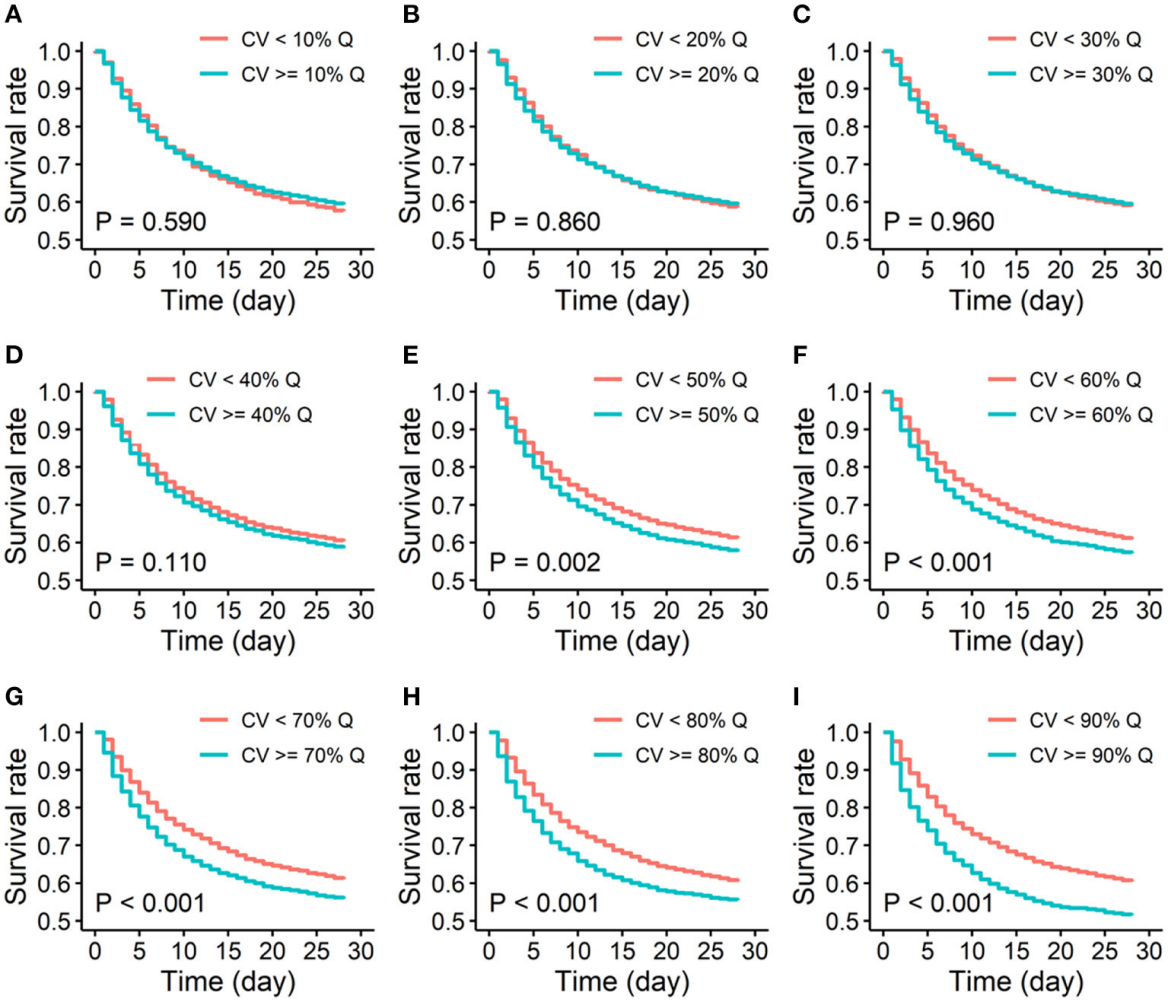
Kaplan-Meier survival curves of low and high variability groups of 28-day mortality. **(A-I)**. Quantiles of MAP variability from 10% to 90% with 10% increasement each were considered to divide study participants into two groups: high variability (≥ each quantile of CV) and low variability (< each quantile of CV). CV: coefficient of variation; % Q: % quantile of CV of MAP.

Furthermore, Kaplan-Meier survival curves of two risk groups stratified by different quantiles of MAP variability were also analyzed. The difference in 28-day survivals between the two groups increased with increasing quantile of MAP variability. 90% quantile of MAP variability as the cutoff reached the largest difference in 28-day survivals (Figure 3). Therefore, 90% quantile of MAP variability was determined as the threshold because it generated the largest odds ratio associated with the increased risk of short-term mortality and the largest difference in 28-day overall survivals.

### Characteristics of study participants in low and high MAP variability subgroups

According to 90% quantile of MAP variability, study participants were classified into two groups: high variability (≥90% quantile of CV) and low variability (< 90% quantile of CV). We further summarized the characteristics of study participants in high and low variability groups.

Among the 1,556 patients with over 90% MAP variability, the median age was 71, which was significantly older than that with < 90% MAP variability (P < 0.001). We also observed that in patients with over 90% MAP variability, there were more women, larger SOFA scores, higher in-hospital and 28-day mortality, lower average SBP, DBP and MAP (*P* < 0.001). Detailed characteristics of study participants are shown in Table 1.

### Association between increased MAP variability and mortality

Study participants were first categorized into low and high variability groups according to 90% quantile of MAP variability. Associations between MAP variability categories and in-hospital mortality, MAP variability categories and 28-day mortality were then carried out using logistic regression and Cox regression models, respectively. The odds ratio for in-hospital mortality was 2.351 (95% CI: 2.064–2.673) and the hazard ratio for 28-day mortality was 2.064 (95% CI: 1.820–2.337). Multivariate logistic regression and Cox regression were further considered to adjust covariates, including age, gender, race, SOFA score and ventilation. The significant associations between MAP variability and risk of in-hospital and 28-day mortality still remained after adjusting covariates (Table 2).

**Table 2:** ORs and HRs associated with in-hospital mortality and 28-day mortality.

**Univariate**	**Hospital**,	**28 d, HR**
	**OR (95% CI)**	**(95% CI)**
	2.351 (2.064–2.673)	2.064 (1.820–2.337)
**Multivariate**
Model 1	2.01 (1.758–2.294)	1.727 (1.516–1.963)
Model 2	1.49 (1.288–1.720)	1.293 (1.124–1.484)
Model 3	1.486 (1.284–1.715)	1.29 (1.122–1.481)

Univariate model: mortality ~ group of MAP variability. Model 1: univariate model adjusted for age, sex and ethnicity. Model 2: Model 1 plus Sequential Organ Failure Assessment score. Model 3: Model 2 plus ventilation. OR, odds ratio; HR, hazard ratio.

Taken together, these results suggested that increased MAP variability (especially over 90% of MAP variability) was associated with increased risk of in-hospital mortality, and 28-day mortality in ICU patients.

### Sensitivity analysis

To exclude the influence of the clinical status severity on the association between increased MAP variability and short-term mortality, we further conducted subgroup analysis using SOFA scores. Patients were divided into low (< 3) and high (≥ 3) SOFA score groups according to the median of the SOFA score. In subgroup analyses, increased MAP variability was also associated with increased risk of in-hospital and 28-day mortality. Specifically, the odds ratio for in-hospital mortality was 1.700 (95% CI, 1.184–2.393) in the low SOFA score group and 1.742 (95% CI, 1.503–2.016) in the high SOFA score group. The hazard ratio for 28-day mortality was 1.338 (95% CI, 0.952–1.847) in the low SOFA score group and 1.534 (95% CI, 1.328–1.769) in the high SOFA score group (Table 3). These results suggested that increased MAP variability is a valuable prognostic factor for short-term mortality independent of SOFA scores.

**Table 3:** ORs and HRs with 95% CIs for mortality in patients with different SOFA scores.

	**Hospital,**	**28 d,**
	**OR (95% CI)**	**HR (95% CI)**
**SOFA** **<** **3 (*****n*** **=** **6,537)**
Univariate	2.5 (1.768–3.460)	2.023 (1.459–2.748)
**Multivariate**
Model 1	1.811 (1.267–2.537)	1.414 (1.009–1.946)
Model 2	1.7 (1.184–2.393)	1.338 (0.952–1.847)
**SOFA** **≥3 (*****n*** **=** **9,023)**
Univariate	1.924 (1.665–2.218)	1.718 (1.493–1.973)
**Multivariate**
Model 1	1.759 (1.518–2.035)	1.547 (1.340–1.784)
Model 2	1.742 (1.503–2.016)	1.534 (1.328–1.769)

Univariate model: mortality ~ group of MAP variability. Model 1: univariate model adjusted for age, sex and ethnicity. Model 2: Model 1 plus ventilation. OR, odds ratio; HR, hazard ratio; SOFA, Sequential Organ Failure Assessment.

## Discussion

In this study, coefficient of variation was applied to describe MAP variability of patients in ICU. Dividing patients into two groups according to different quantiles of MAP variability, 90% was the one that yielded the largest odds ratio associated with the increased risk of short-term mortality. Applying logistic regression and Cox regression models, we further confirmed that increased variability of MAP (especially over 90% of MAP variability) was associated with increased risk of short-term mortality. The results remained stable in patients with different SOFA scores. These findings suggest that increased variability of MAP may aid in the early identification of critically ill patients with a high risk of mortality.

As one of the most routinely measured physiological variables in clinical setting, many studies have explored the relationship between blood pressure variability and mortality from both prognostic significance and treatment perspectives (10, 24, 25). The increased variation of BP has been reported to be associated with various organ injuries, high risks of cardiovascular and cerebrovascular events, and mortality (6–9, 26). Some studies reported that blood pressure reverse dipping may be associated with cardiovascular events in patients with essential hypertension (27–30). In critical ill patients, decrease (between −5 and 5%) in mean arterial pressure (MAP) fluctuation calculated by (nighttime MAP–daytime MAP)/24-h MAP may be related to adverse outcomes in critically ill patients (31). However, which thresholds of MAP variability are clinically relevant remains unclear.

Blood pressure variability have been quantified by several measurements such as standard deviation, coefficient of variation, and average real variability (i.e., the average of the absolute differences between consecutive measurements) (32). Although previous studies have hinted prognostic significance and therapeutic promise of blood pressure variability, their applications were rare in critical care patients. A recent study investigated the relationship between blood pressure variability and short-term mortality, where blood pressure variability was quantified by coefficient of variation and average real variability, considered as both continuous and categorical variable. However, when dividing subjects equally into 4 groups, the association between continuous blood pressure variability and short-term mortality was weak, near but not reaching statistical significance (*p* < 0.05). The association between categorical blood pressure variability and short-term mortality reached statistical significance when blood pressure variability fell into the 4th quartile (i.e., 75% quantile) (13). Their findings suggested that further exploration about the threshold of blood pressure variability associated with short-term mortality is necessary.

To our knowledge, our study is the first to compare different thresholds of MAP variability such that increased MAP variability is associated with short-term mortality. The odds ratio increased with the increasing quantile of MAP variability. Our study showed that 4th quartile may not be the optimal threshold in clinical settings. The top 10% of patients with the highest blood pressure variability were showed to have a much higher risks of short-term mortality. This subset of patients with extremely high MAP variability may lose physiological homeostatic regulation and circadian rhythms of blood pressure, thereby leading to target organ damage and subsequent cardiovascular events, resulting in increased risk of short-term morality in the ICU. Preclinical studies have demonstrated that calcium channel blockers such as amlodipine can alleviate excessive blood pressure fluctuations and reduce cardiovascular morbidity and mortality in hypertensive patients (33). Therefore, early diagnosis of these ICU patients with extremely high MAP variability, especially those with underlying diseases (such as hypertension, diabetes mellitus, chronic kidney disease, etc.), and timely administration of drugs that reduce MAP variability is expected to reduce short-term mortality in the ICU. Our proposed MAP variability threshold could serve as such a tool for early identification of critically ill patients at high risk of short-term mortality in the ICU.

## Limitation

Several potential limitations should be acknowledged in our study. First, our study is a retrospective study based on a publicly accessible database (MIMIC). Therefore, it was limited to knowledge on BP monitoring device, BP measurements (e.g., automatic or manual), mortality cause and so on. The blood pressure records in the database are not continuous. The time interval between each two successive blood pressure recording may vary. Second, based on our data, the optimal threshold of MAP variability for in-hospital mortality and 28-day mortality was determined to be approximately 90%. However, this threshold needs to be further validated in large independent cohorts before clinical application. Third, this study was data-driven and limited by its applicability. The data used in this study were previously collected from a single-center database MIMIC-III. In future, a well-designed prospective study should be conducted to evaluate causality between BP variability and short-term mortality in multiple clinical centers.

## Conclusion

Increased MAP variability, especially over 90% of MAP variability, is associated with short-term mortality. Our proposed threshold of MAP variability may aid in the early identification of critically ill patients with a high risk of short-term mortality.

## Data availability statement

The raw data supporting the conclusions of this article will be made available by the authors, without undue reservation.

## Ethics statement

The studies involving human participants were reviewed and approved by Institutional Review Boards of Beth Israel Deaconess Medical Center (Boston, MA) and the Massachusetts Institute of Technology (Cambridge, MA). Written informed consent for participation was not required for this study in accordance with the national legislation and the institutional requirements.

## Author contributions

JY, DL, and YY analyzed the data. YL, PL, and XP designed the study. JY, DL, PL, and XP drafted the paper. All authors edited and approved the paper.

## Funding

This work was supported by the Natural Science Foundation of Shanghai under grant 20JC1413800.

## Conflict of interest

The authors declare that the research was conducted in the absence of any commercial or financial relationships that could be construed as a potential conflict of interest.

## Publisher's note

All claims expressed in this article are solely those of the authors and do not necessarily represent those of their affiliated organizations, or those of the publisher, the editors and the reviewers. Any product that may be evaluated in this article, or claim that may be made by its manufacturer, is not guaranteed or endorsed by the publisher.
