# Supplementary material for: Increased variability of mean arterial pressure is associated with increased risk of short-term mortality in intensive care unit: A retrospective study
Source: Front Neurol. 2022 Sep 29;13:999540. doi: 10.3389/fneur.2022.999540 (PMC9557059; doi:10.3389/fneur.2022.999540)
Supplement: Supplementary file 1 [file Data_Sheet_1.PDF]

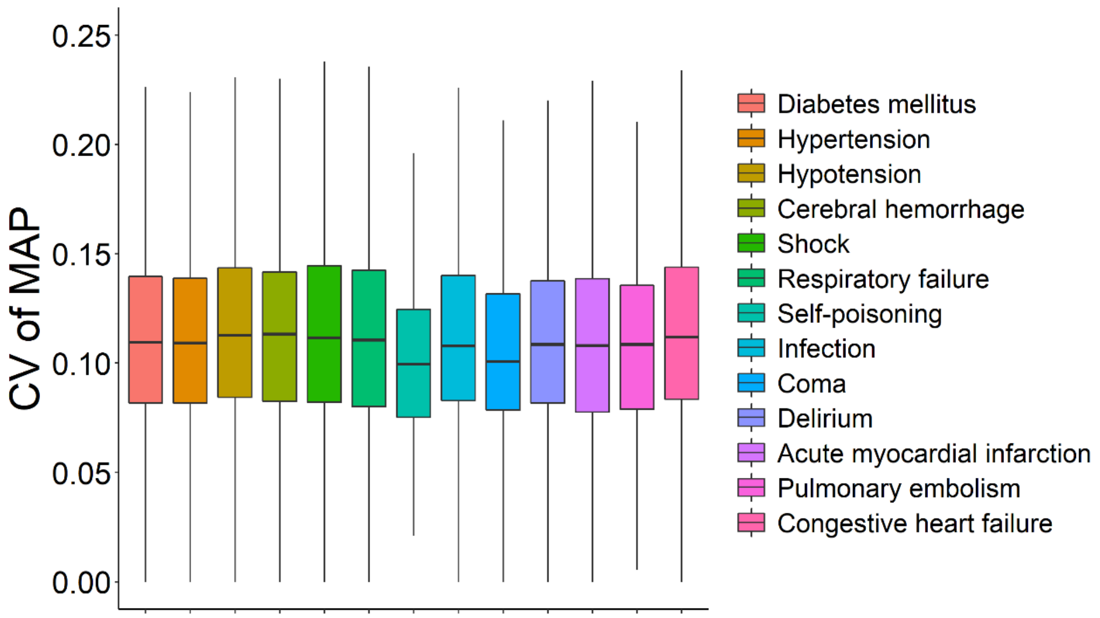

Supplemental Figure S1. Coefficient of variation of MAP. Study participants were divided into subgroups according to diagnosed diseases at admission. MAP: mean arterial pressure; CV: coefficient of variation
